# Supplementary material for: Social Inequalities in Environmental Noise Exposure: A Review of Evidence in the WHO European Region
Source: Int J Environ Res Public Health. 2019 Mar 20;16(6):1011. doi: 10.3390/ijerph16061011 (PMC6466273; doi:10.3390/ijerph16061011)
Supplement: Supplementary file 1 [file ijerph-16-01011-s001.pdf]

Supplement S1: Search strategy

Web of Science - Advanced Search

TS=(disadvantaged OR disadvantage OR deprived OR social OR socio* OR vulnerable OR vulnerability OR psychosocial OR psycho-social OR socio-economic OR deprivation OR socio-demographic)

AND

TS=(noise)

AND

TS=(inequality OR inequity OR inequities OR inequalities OR unequal OR “environmental justice” OR “environmental injustice”)

AND

PY=(2010-2017)

Restrict results by language and document types (to be selected manually)

- English
- Article

Scopus - Advanced Search

ALL(disadvantaged OR disadvantage OR deprived OR social OR socio* OR vulnerable OR vulnerability OR psychosocial OR psycho-social OR socio-economic OR deprivation OR socio-demographic) AND TITLE-ABS-KEY(noise) AND TITLE-ABS-KEY(inequality OR inequity OR inequities OR inequalities OR unequal OR "environmental justice" OR "environmental injustice") AND LANGUAGE(english) AND PUBYEAR > 2009 AND PUBYEAR < 2018 AND DOCTYPE(ar) AND NOT INDEX (medline)

Supplement S2: PRISMA checklist

Section/topic	#	Checklist item	Reported on page #
**TITLE**			
Title	1	Identify the report as a systematic review, meta-analysis, or both.	1
**ABSTRACT**			
Structured summary	2	Provide a structured summary including, as applicable: background; objectives; data sources; study eligibility criteria, participants, and interventions; study appraisal and synthesis methods; results; limitations; conclusions and implications of key findings; systematic review registration number.	1
**INTRODUCTION**			
Rationale	3	Describe the rationale for the review in the context of what is already known.	1-2
Objectives	4	Provide an explicit statement of questions being addressed with reference to participants, interventions, comparisons, outcomes, and study design (PICOS).	2
**METHODS**			
Protocol and registration	5	Indicate if a review protocol exists, if and where it can be accessed (e.g., Web address), and, if available, provide registration information including registration number.	2-3
Eligibility criteria	6	Specify study characteristics (e.g., PICOS, length of follow-up) and report characteristics (e.g., years considered, language, publication status) used as criteria for eligibility, giving rationale.	3-4
Information sources	7	Describe all information sources (e.g., databases with dates of coverage, contact with study authors to identify additional studies) in the search and date last searched.	3
Search	8	Present full electronic search strategy for at least one database, including any limits used, such that it could be repeated.	3 and S1
Study selection	9	State the process for selecting studies (i.e., screening, eligibility, included in systematic review, and, if applicable, included in the meta-analysis).	3-4
Data collection process	10	Describe method of data extraction from reports (e.g., piloted forms, independently, in duplicate) and any processes for obtaining and confirming data from investigators.	4-5 and S3
Data items	11	List and define all variables for which data were sought (e.g., PICOS, funding sources) and any assumptions and simplifications made.	4-5

Risk of bias in individual studies	12	Describe methods used for assessing risk of bias of individual studies (including specification of whether this was done at the study or outcome level), and how this information is to be used in any data synthesis.	5
Summary measures	13	State the principal summary measures (e.g., risk ratio, difference in means).	n.a.
Synthesis of results	14	Describe the methods of handling data and combining results of studies, if done, including measures of consistency (e.g., I^2) for each meta-analysis.	n.a.

Section/topic	#	Checklist item	Reported on page #
Risk of bias across studies	15	Specify any assessment of risk of bias that may affect the cumulative evidence (e.g., publication bias, selective reporting within studies).	n.a.
Additional analyses	16	Describe methods of additional analyses (e.g., sensitivity or subgroup analyses, meta-regression), if done, indicating which were pre-specified.	n.a.
**RESULTS**			
Study selection	17	Give numbers of studies screened, assessed for eligibility, and included in the review, with reasons for exclusions at each stage, ideally with a flow diagram.	5-6
Study characteristics	18	For each study, present characteristics for which data were extracted (e.g., study size, PICOS, follow-up period) and provide the citations.	6-10, S3
Risk of bias within studies	19	Present data on risk of bias of each study and, if available, any outcome level assessment (see item 12).	n.a.
Results of individual studies	20	For all outcomes considered (benefits or harms), present, for each study: (a) simple summary data for each intervention group (b) effect estimates and confidence intervals, ideally with a forest plot.	n.a.
Synthesis of results	21	Present results of each meta-analysis done, including confidence intervals and measures of consistency.	n.a.
Risk of bias across studies	22	Present results of any assessment of risk of bias across studies (see Item 15).	n.a.
Additional analysis	23	Give results of additional analyses, if done (e.g., sensitivity or subgroup analyses, meta-regression [see Item 16]).	n.a.
**DISCUSSION**			
Summary of evidence	24	Summarize the main findings including the strength of evidence for each main outcome; consider their relevance to key groups (e.g., healthcare providers, users, and policy makers).	11
Limitations	25	Discuss limitations at study and outcome level (e.g., risk of bias), and at review-level (e.g., incomplete retrieval of identified research, reporting bias).	14

Conclusions	26	Provide a general interpretation of the results in the context of other evidence, and implications for future research.	11-14
**FUNDING**			
Funding	27	Describe sources of funding for the systematic review and other support (e.g., supply of data); role of funders for the systematic review.	15

From: Moher D, Liberati A, Tetzlaff J, Altman DG, The PRISMA Group (2009). Preferred Reporting Items for Systematic Reviews and Meta-Analyses: The PRISMA Statement. PLoS Med 6(7): e1000097. doi:10.1371/journal.pmed1000097

For more information, visit: **www.prisma-statement.org**.

S3: Data extraction of inequalities in environmental noise.

**Author and Years**	**Place of Study**	**Unit of Analysis (Study Population and Sample Size)**	**Study Type**	**Measurement and Operationalisation of Noise**	**Sociodemographic and Socioeconomic Characteristics (Framework: PROGRESS-PLUS) Analysed**	**Environmental Inequality Analysis**	**Results on Environmental Inequalities in Symbols**
Bocquier et al., 2013 [29]	Marseilles, France	**Aggregated level:** 338 census blocks	Ecological study	**Objective:** Noise Map **Operationalisation:** Road potential noise exposure indicator as day-evening-night level in decibels (dB) (L_{den})	Quintiles of deprivation index based on census data including job, income, educational level, housing, household type, immigration status	**Bivariate analysis:** Spearman´s correlation coefficient (Scatter plot)<br><br>Regression models:<br>1.Bivariate analysis<br>2. ordinary least square regression model (OLS) (non-linear relationship<br>3. Spatial models which considered spatial autocorrelation. (same association: non-linear relationship, too)	**Bivariate analysis:**<br>*Potential noise exposure indicator Lden*<br>Spearman correlation:<br>⊕ High deprivation (n.l. in middle groups))<br>OLS model:<br>⊕ high deprivation (n.l. in middle groups)<br>Simultaneous autoregressive model including the response variable as a covariate in the form of a spatially lagged variable:<br>⊕ high deprivation (n.l. in middle groups)<br>Simultaneous autoregressive model including a spatial error structure:<br>⊕ high deprivation (n.l. in middle groups)<br>Intrinsic conditional autoregressive model:<br>⊕ high deprivation (n.l. in middle groups)
Flacke et al., 2016 [31]	Dortmund, Germany	**Aggregated level:** 170 neighbourhoods	Ecological study	**Objective:** Noise map **Operationalisation:** Share of area having noise impact > 55 Decibels A weighted dB(A) Lden as a % of the total area of the neighbourhood	Socio-economic disadvantage: Percentage of inhabitants receiving unemployment benefits or social welfare aids as a % of the total population	**Bivariate analysis:** Spearman´s correlation coefficient	n.s. socio-economic disadvantage
Grelat et al., 2016 [27]	Besançon, France	**Individual level:** 517 children, 46,3% boys, 53,7% girls	Cross-sectional	**Subjective:** Questionnaire **Operationalisation:** Dichotomized: not annoyed (Likert scale = not at all or a little) or annoyed (Likert-scale= moderately or very much)	Secondary Data: Urban environment, Percentage of households without a car (for an increase of 10%)<br>Questionnaire: Household socio economic status	**Multivariate Analysis:** Logistic regression (road traffic annoyance adjusted for: satisfaction with the environment, outdoor noise level in front of child's	**Multivariate Analysis**<br>*Road traffic annoyance*<br>⊕ 100% public/social housing in neighbourhood<br>*General transportation noise annoyance*<br>⊕ low household socioeconomic status (n.l. in middle group)<br>*Ambient noise annoyance*

						bedroom, urban environment; general transportation noise annoyance and ambient noise annoyance adjusted for: satisfaction with the dwelling, missing data, outdoor noise level in front of child's bedroom	⊕ High percentage of households without a car
Havard et al., 2011 [15]	Paris, France	**Individual level:** N=2130 in 571 neighbourhoods	Cross-sectional	**Objective:** Noise Map<br>**Operationalisation:** Exposure to road traffic noise within 250m radius buffer on exact residential building, averaging calculations points included within the buffer	<u>Individual level</u><br>- country of citizenship (ref. French citizen)<br>- Human Development Index (HDI) of country of citizenship (ref. French HDI)<br><u>Neighbourhood level</u><br>- Proportion of highly educated residents<br>- mean value of dwellings<br>- neighbourhood proportion of non-French citizens<br>- proportion of citizens from low HDI countries<br>- proportion of citizens from medium HDI countries<br>- proportion of citizens from high HDI countries	**Bivariate analysis:** Analysis of variance with Jonckheere-Terpstra test for trend<br>**Multivariate analysis:**<br>- Linear regression model<br>- Multilevel linear regression model<br>- spatial multilevel linear regression model<br>- standard multilevel regression model	**Bivariate:**<br><u>*dB(A)*</u><br>⊖ low neighbourhood proportion of highly educated residents; low mean value of dwellings<br>⊕ high neighbourhood proportion of non-French citizens (n.l. for middle group)<br>**Mulitvariate:**<br>*Exposure to road traffic noise*<br><u>Linear regression model:</u><br>⊕ non-French citizenship; high neighbourhood proportion of non-French citizens<br>⊖ low neighbourhood proportion of highly educated people; low mean value of dwellings<br><u>Multilevel linear regression model:</u><br>⊕ non-French citizenship; high neighbourhood proportion of non-French citizens<br>⊖ low neighbourhood proportion of highly educated people; low mean value of dwellings<br><u>Spatial multilevel linear regression model:</u><br>⊕ Non-French citizenship<br>n.s. low neighbourhood proportion of highly educated residents; mean value of dwelling; high proportion of non-French citizens<br><u>Standard multilevel regression model</u>

							⊖ high neighbourhood proportion of citizens from low human development index countries;<br>n.s. individual human development index of citizenship; neighbourhood proportion of highly educated people; neighbourhood mean value of dwellings; neighbourhood proportion of citizens from low human development index countries;
Lakes et al., 2014 [32]	Berlin, Germany	**Aggregated level:** Planning Units	Ecological study	**Objective:** Noise Map<br>**Operationalisation:** Average weighted sound pressure level L_n in the residual area for each planning unit (Average Ln R)	Socio-economic indicator (index)	**Bivariate analysis:** Pearsons's correlation coefficient	n.s. socio-economic disadvantage
Méline et al., 2013 [28]	Ille-de-France region, France	**Individual level:** Adults (N=7290) from the RECORD cohort	Cross-sectional	**Objective:** Noise Map<br>**Operationalisation:**<br>Percentiles of road traffic noise at the place of residence (with L_{den} indicator and in dB(A)):<br>- road traffic noise at the place of residence (with the Lden indicator and in dB(A)<br>- road traffic noise at the 25th percentile of 500m radius street network buffers around the place of residence (with the Lden indicator and in dB(A)<br>- road traffic noise at the median of 500m radius street network buffers around the place of residence (with Lden indicator and in dB(A)<br>- road traffic noise at the 75th percentile of 500m radius street network buffers around the place of residence (with the Lden indicator) in dB(A)<br><br>**Subjective:** Questionnaire<br>**Operationalisation:** Annoyance due to road traffic noise	**Aggregated level:** neighbourhood proportion of highly educated residents, neighbourhood median income in 500m street networks buffer around the place of residence,<br>**Individual Level:** gender, age, education, household income, non-ownership of dwelling	**Bivariate analysis:** Analysis of variance with Jonckheere-Terpstra test for trend	**Objective:**<br>**Descriptive**<br>*road traffic noise at the place of residence*<br>⊕ low proportion of highly educated residents in neighbourhood<br>*road traffic noise at the 25th percentile of 500m*<br>⊕ low proportion of highly educated residents in neighbourhood<br>*road traffic noise at the median of 500m radius street network buffers*<br>⊕ low proportion of highly educated residents in neighbourhood<br>*road traffic noise at the 75th percentile of 500m radius street network buffers*<br>⊖ low proportion of highly educated residents in neighbourhood<br>**Subjective:**<br>*Annoyance due to road traffic noise:*<br>**Multivariate**<br>⊕ nonownership of dwelling, low education (ns. For middle-high category); low household income; low neighbourhood median income<br>n.s.: sex; age

Riedel et al., 2014 [29]	Ruhr-Area, Germany	**Individual level:** N=1191 DHS dataset)+<br>N=269 (SAVE dataset)<br>Total: 1460 adults	Cross-sectional	**Objective:** Noise Map<br>**Operationalisation in descriptive analysis:** Exposed to road traffic noise > 55dB (A) L_{den} compared to exposed to road traffic noise ≤ 55dB (A) L_{den})<br>**Operationalisation in multivariate analysis:** subjective noise annoyance	Gender, age, migration background, education	**Bivariate:** Chi2 test<br>**Multivariate:** Multivariate linear regression and multivariate logistic regression (adjusted for objective noise exposure, health related factors, attitude towards silence)	**Bivariate:**<br>DHS<br>*L_{den}*<br>⊖ older age[1]<br>n.s. sex, migration background; education<br>SAVE<br>⊕ low education<br>n.s. sex; age; migration background<br>**Multivariate linear regression:**<br>*Noise annoyance*<br>DHS<br>n.s.: sex; age; migration background; education<br>SAVE<br>⊕ migration background<br>n.s.: sex; age; education<br>**Multivariate logistic regression:**<br>*Noise annoyance*<br>DHS<br>n.s. sex; age; migration background; education<br>SAVE<br>n.s. sex; age; migration background; education
Xie and Kang, 2010 [33]	Greater London, England	**Aggregated level:** On random number generation 329 postcodes from Greater London were selected	Ecological study	**Objective:** Noise map data from 2004 to 2008; average noise levels at heights of 4m above local ground level; $L_{den,}$<br>**Operationalisation:**<br>Average noise level (L_{ave}) and intrusive noise level, representing the level of noise exceeded for 10% of the specified measurement period (L_{10})	Neighbourhood level:<br>total deprivation index, living environment deprivation index, income deprivation index, employment deprivation index, education deprivation index,<br>At borough level:<br>age, income, income	**Bivariate:** Spearman correlation for analyses at the neighbourhood level and Person correlation for most analyses at the borough level, except where indicated	**Bivariate:**<br>At neighbourhood level<br>*L_{ave}*<br>⊕ high total deprivation; high living environment deprivation;<br>n.s. income deprivation; employment deprivation; education deprivation; % of people being disabled; old age<br>*L_{10}*<br>⊕ high total deprivation; high living environment deprivation;<br>n.s. income deprivation; employment deprivation; education deprivation; % of people being disabled; old age, employment rates<br>At borough level<br>*L_{ave}*

							⊖ low median income<br>n.s.:age<br>*L_{10}*<br>⊖ low median income<br>n.s.: age

n.s. = not significant
„ ⊕ " = supports our hypothesis: low SEP groups have higher noise exposure compared to high SEP groups or higher SEP groups have lower noise exposure compared to lower SEP groups / significant association in correlation or multivariate analysis (p-value < 0.05)
„⊖" = challenges our hypothesis: low SEP groups have lower noise exposure compared to high SEP groups or higher SEP groups have higher noise exposure compared to lower SEP groups / significant association in correlation or multivariate analysis (p-value < 0.05)
[1)] old people are defined as lower SEP group
